# Combined effects of lung disease history, environmental exposures, and family history of lung cancer to susceptibility of lung cancer in Chinese non-smokers

**DOI:** 10.1186/s12931-021-01802-z

**Published:** 2021-07-23

**Authors:** Fanglin Yu, Rendong Xiao, Xu Li, Zhijian Hu, Lin Cai, Fei He

**Affiliations:** 1 Experiment Center, School of Public Health, Fujian Medical University, Fuzhou, Fujian 350122 People’s Republic of China; 2 Department of Thoracic Surgery, First Affiliated Hospital of Fujian Medical University, Fuzhou, 350005 Fujian People’s Republic of China; 3 Department of Epidemiology and Health Statistics, School of Public Health, Fujian Medical University, Fuzhou, 350122 Fujian People’s Republic of China; 4 Fujian Provincial Key Laboratory of Environment Factors and Cancer, Fujian Medical University, Fuzhou, 350122 Fujian People’s Republic of China; 5 Key Laboratory of Ministry of Education for Gastrointestinal Cancer, Fujian Medical University, Fuzhou, 350122 People’s Republic of China

**Keywords:** Lung cancer, Lung disease history, Family history of lung cancer, Environmental exposure index

## Abstract

**Background:**

Although cigarette smoking is a major risk factor for lung cancer, the incidence rate of lung cancer among non-smokers is notable. The etiology and potential mechanism of non-smoker lung cancer are worthy of further research. This study was designed to explore the collective effects of environmental factors and the relationship between environmental exposure index (EEI) and lung cancer among non-smokers by evaluating the joint effects among lung disease history, environmental factors, and family history of lung cancer without smoking confounders.

**Methods:**

A total of 767 never-smoked lung cancer cases and 767 sex- and age-matched controls were selected from the department of Thoracic Surgery and Respiratory Medicine of three hospitals in Fujian, China. We used two methods to develop the EEI according to 12 statistically significant environmental risk factors. Restricted cubic spline (RCS) was applied to analyze the non-linear relationship between EEI and lung cancer in non-smokers. Combined effects, additive interaction, and multiplicative interaction were assessed among lung disease history, EEI, and family history of lung cancer to estimate susceptibility to develop lung cancer.

**Results:**

Lung disease history, especially asthma, was significantly associated with an increased risk of lung cancer with an odds ratio (OR) for asthma history of 14.720 (95% CI: 1.877–115.449). Family history of lung cancer was related to susceptibility of lung cancer (OR = 3.347, 95% CI: 1.930–5.806). According to type of relatives and cancer, a parental or children’s history and a sibling’s history of lung cancer were significantly associated with an increased risk of lung cancer. The positive association between EEI and lung cancer was apparently stronger in those with lung disease history or family lung cancer history. Furthermore, there was a addictive interaction between EEI and lung disease history, and a possibly addictive interaction between EEI and family lung cancer history on development of lung cancer.

**Conclusions:**

There were combined effects among lung disease history, environmental exposures, and family history of lung cancer toward susceptibility to lung cancer in Chinese non-smokers. Non-smokers who had a family history of lung cancer were at higher risk of lung cancer than non-smokers who had lung disease history. Non-smokers with family cancer history may obtain benefits from removal of environmental exposures and active treatment of lung disease.

**Supplementary Information:**

The online version contains supplementary material available at 10.1186/s12931-021-01802-z.

## Introduction

Worldwide, lung cancer was the second leading cause of cancer incidence with 2,206,771 newly diagnosed cancer cases and the first leading cause of cancer mortality with 1,796,144 deaths predicted in 2020, accounting for 11.4% of the total new cases and 18% of cancer deaths, respectively [1]. Data published in 2021 in China indicate that there were 815,563 new lung cancer cases and 714,699 new lung cancer deaths in 2020, both exceeded one-third of the number in the world, ranking second for cancer incidence and first for cancer death [2].

Since the 1960s, many studies on the etiology of lung cancer have accumulated evidence to indicate that smoking is an important risk for lung cancer [3]. However, about 10–15% of lung cancer patients of Western countries are non-smokers, yet non-smokers account for as much as 30–40% of the patients in Asian countries [4, 5]. In Asians, a significantly increased incidence of lung cancer in non-smokers was noted in a study spanning three decades from 1970 to 2000, which report from 15.9 to 32.8% [6]. The California teachers’ study cohort [7] reported that the age-adjusted incidence rate of lung cancer was 4.8/100,000 in non-smoking men and 20.8/100,000 in non-smoking women, yet paradoxically, non-smoking women are more likely to develop lung cancer than men. Some studies suggest that non-smoker lung cancer is a separate category of cancer, yet is significant as the seventh most common tumor mortality worldwide [8]; therefore, the etiology and potential mechanism of non-smoker lung cancer have become areas of concern for cancer researchers.

At present, the related risk factors for lung cancer in non-smokers are exposure to environmental tobacco smoke (ETS), cooking oil fumes (COF), residential radon, particulate matter 2.5 (PM_2.5_), and other air pollution-related particulate matter exposures [9–11]. The complex pathogenesis of lung cancer is thought to be related to the interaction between genetic and environmental factors. Similar environmental factors and genetic variations may contribute to the familial aggregation of lung cancer in first-degree relatives [12, 13]. Recent studies have concluded that previous lung diseases may be linked to lung carcinogenesis in many different populations [14–16]. It was reported that in Taiwanese, asthma, chronic obstructive pulmonary disease (COPD), and tuberculosis (TB) were associated with an increased risk of all major subtypes of lung cancer and the risk was the highest among women with TB. Most notably, susceptibility to lung cancer was higher in patients with coexistence of pulmonary diseases [17, 18].

If the associations of lung cancer with lung disease history and other factors, such as family lung cancer history and environmental factors are present, other factors could be confounding factors in addressing the association with lung disease history and vice versa. However, few such studies have been described. For a detailed evaluation of the joint effects among lung disease history, environmental factors, and family history of lung cancer without smoking confounders, we conducted a hospital-based case–control study of Chinese non-smokers to explore the relationships among all genetic and environmental factors.

## Materials and methods

### Subjects

A hospital-based case–control study was designed using patient data selected from the departments of Thoracic Surgery and Respiratory Medicine of three hospitals, the First Affiliated Hospital of Fujian Medical University, the Affiliated Union Hospital of Fujian Medical University, and the Fuzhou General Hospital of Nanjing Military Region. The inclusion criteria of participants were (1) newly diagnosed and confirmed by bronchoscope examination and pathological test, (2) from January 2006 to June 2015, (3) local residence in Fujian for more than 10 years, (4) no smoking history (cumulative smoking less than 100 cigarettes), and exclusion criteria were pathological diagnosis of benign lesions, secondary lung cancer, and inability to answer questions clearly. Healthy controls were selected from hospital visitors and the community population and were matched to the cases on age (± 3 years) and sex in the same period. The inclusion criteria of healthy controls were as follows: (1) no history of tumor disease, (2) no family members of lung cancer case group, and (3) no smoking history (cumulative smoking less than 100 cigarettes). This study was approved by the Ethics Committee of Fujian Medical University and all the interviewees gave informed consent in compliance with the Declaration of Helsinki.

### Data collection and variable definition

A unified structured questionnaire was used to survey all persons by means of face-to-face interviews conducted by trained investigators. The survey included sociodemographic characteristics, polluting companies near home, irritant smell after renovations, use of a range hood, oil temperature when cooking, frying by yourself, habit of eating garlic raw, food temperature when eating, consumption of food (green vegetable, fruit, meat, fish, seafood, dairy products, bean products, eggs, pickled food, fried food, smoked food), vitamin intake, drinking history, tea drinking history, environmental tobacco smoke (ETS), physical activity, lung disease history, and family cancer history. Food intake was investigated for the quality and frequency of vegetables and meat. Drinking alcohol is defined as at least 1 time/week and for more than half a year. Drinking tea is defined as at least 1 cup/week and for more than half a year. ETS is defined as non-smokers smoke at least one day (more than 15 min per day) of inhaled cigarettes or smoke exhaled by smokers. Lung disease history was self-reported for the period of one year before interviews. The information on family cancer history included the number, sex, cancer type, and specific kinship of biological relatives that had ever been diagnosed with cancer. A first-degree relative [19] is defined as biological parents, siblings, and children (Additional file 3).

### Statistical analysis

The chi-squared test and t-test were used to analyze the general demographic characteristics of the patients and control groups. The unconditional logistic regression model was used to analyze the associations between variables and lung cancer and provide estimated odds ratios (ORs) and the 95% confidence interval (CI) for the effects of risk factors.

The statistically significant variables obtained from multivariable-adjusted logistic regression model were given an exposure weight. We used two methods to calculate the environmental exposure index (EEI). (1) EEI-1: According to the method described by Katsouyanni et al.[20], we assigned the weight of excess OR (OR-1) to subjects at high risk of lung cancer regarding selected environmental risk factors from this study and assigned “0” to those without high risk. (2) EEI-2: We assigned a particular weight that was equal to 10 × β coefficient [21] and rounded to the closest integer for individuals at high risk of lung cancer regarding selected environmental risk factors from this study and assigned “0” to those without high risk. For either method, the EEI was calculated by summing significant variable weights.

The difference in the score of EEI between cases and matched controls was compared by t-test. Restricted cubic spline (RCS) was applied to analyze the non-linear relationship between EEI and lung cancer in non-smokers. We used the criterion that β (except β_1_) is equal to 0 to test whether the linear hypothesis test is rejected. We then used the tertile scores of EEI as the cut-off point for all subjects.

Combined effects and multiplicative interaction of lung disease history, environmental exposures, and family history of lung cancer were considered as factors linked to susceptibility to lung cancer and were assessed by logistic regression model. The Hosmer and Lemeshow methods [22] were used to construct the cooperative index to quantify the potential additive interactions between EEI and family history of lung cancer, EEI and lung disease history, family history of lung cancer and lung disease history. If there was no additive interaction between the two factors, the confidence intervals of the relative excess risk due to interaction (RERI) and the attributable proportion due to interaction (AP) should contain 0, and the confidence interval of the synergy index (S) should contain 1.

Statistical analyzes were performed using the SPSS version 25.0 (IBM Corporation, Armonk, New York, USA) and STATA version 15.0 (Stata Corp, College Station, Texas, USA) statistical software packages. *P* values of the all results were calculated as two-tailed tests with a level α = 0.05.

## Results

### General demographic characteristics of the non-smoker

A total of 767 never-smoked lung cancer cases and 767 never-smoked community controls who had complete information were included. Slightly more cases than controls had low level education and were manual workers or were classified as occupation unknown (Table 1).

**Table 1:** The distribution of demographic variables among the never smoking cases and controls

Demographic variables	Controls N (%)	Cases N (%)	*χ*^*2*^	*p*
Sex				
Male	153 (19.9%)	153 (19.9%)		
Female	614 (80.1%)	614 (80.1%)		
Age (years)			0.066	0.967
50	202 (26.3%)	205 (26.7%)		
50–65	376 (49.0%)	377 (49.2%)		
65	189 (24.6%)	185 (24.1%)		
Marriage			1.337	0.248
Married	704 (91.8%)	691 (90.1%)		
Unmarried/divorced	63 (8.2%)	76 (9.9%)		
Education			82.993	< 0.001
Primary school and below	260 (33.9%)	424 (55.3%)		
Middle school	328 (42.8%)	261 (34.0%)		
College and above	179 (23.3%)	82 (10.7%)		
Occupation			74.868	< 0.001
Mental worker	367 (47.8%)	215 (28.0%)		
Manual worker	324 (42.2%)	395 (51.5%)		
Unemployed / unknown	76 (9.9%)	157 (20.5%)		

Association between family cancer history, lung disease history, and susceptibility to lung cancer among non-smokers.

The distributions of type of family cancer history and lung disease history were all significantly different between cases and controls. The OR for family history of lung cancer derived from logistic regression model was 3.347 (95%CI: 1.930–5.806), whereas no statistical significance was observed for those with family history of other cancers. The OR for lung disease history was 2.041 (95% CI: 1.356–3.071), which tended to be lower for individuals with family history of lung cancer. The OR for asthma history derived from logistic regression model was 14.720 (95% CI: 1.877–115.449); however, no statistical significance was observed for those with other lung disease history (Table 2).

**Table 2:** Distributions of family cancer history and lung disease history by status of cases and controls, the corresponding ORs and 95% CIs among non-smokers

Levels of exposure	Cases	Controls	*p*	OR and 95% CI*
	N (%) (n = 767)	N(%) (n = 767)		
Family cancer history				
Overall cancers	162 (21.1%)	169 (22.0%)	0.185	1.189 (0.921, 1.535)
Type			< 0.001	
Lung cancer	20 (2.6%)	54 (7.0%)	< 0.001	**3.347 (1.930, 5.806)**
Other cancers	142 (18.5%)	115 (15.0%)	0.509	0.909 (0.685, 1.206)
Relationship			0.148	
First-degree relatives	133 (17.3%)	147 (19.2%)	0.054	1.283 (0.995, 1.654)
Other relatives	29 (3.8%)	22 (2.9%)	0.880	0.958 (0.550, 1.669)
Type of cancer and relatives			0.002	
Lung cancer in father, mother or children	13 (1.7%)	28 (3.7%)	0.006	**2.646 (1.323, 5.291)**
Lung cancer in siblings	4 (0.5%)	20 (2.6%)	0.001	**6.329 (2.058, 19.467)**
Other cancers in father, mother or children	81 (10.6%)	73 (9.5%)	0.767	0.948 (0.668, 1.347)
Other cancers in siblings	35 (4.6%)	26 (3.4%)	0.558	0.850 (0.494, 1.463)
Lung cancer in other relatives	3 (0.4%)	6 (0.8%)	0.166	2.804 (0.651, 12.0738)
Other cancers in other relatives	26 (3.4%)	16 (2.1%)	0.538	0.812 (0.417, 1.578)
Lung disease history				
Overall lung disease	44 (5.7%)	73 (9.5%)	0.001	**2.041 (1.356, 3.071)**
Type			0.018	
Chronic bronchitis	19 (2.5%)	23 (3.0%)	0.203	1.523 (0.797, 2.908)
Tuberculosis	10 (1.3%)	16 (2.1%)	0.056	2.256 (0.980, 5.194)
Pneumonia	8 (1.0%)	15 (2.0%)	0.127	2.014 (0.820, 4.946)
Asthma	1 (0.8%)	13 (1.7%)	0.010	**14.720 (1.877, 115.449)**
Other	6 (0.1%)	6 (0.8%)	0.785	1.177 (0.365, 3.793)

The bold values represent *P* values less than 0.05

*OR* odds ratio, *CI* confidence interval

*Adjusted for sex, age, marriage, education, and occupation

### Development of the collective EEI and restricted cubic spline analysis

Overall, the EEI-1 ranged from 0 to 12.318 (median = 3.346) and the mean EEI-1 of cases differed with that of controls (4.177 ± 1.690 vs. 2.688 ± 1.425, *p* < 0.001), whereas the EEI-2 ranged from 0 to 66 (median = 23) and the mean EEI-2 of never-smoked groups also differed between the two groups (28.00 ± 9.73 vs. 18.63 ± 9.16, *p* < 0.001, Table 3). We classified the EEI score into three categories by tertiles for non-smokers (EEI-1: < 2.519, 2.519–3.921, and > 3.921; EEI-2: < 18, 18–27, and > 27). A restricted cubic spline model using four knots located at the 10^th^, 33^rd^, 67^th^, and 90th percentiles of the empirical distributions formed by value of EEI-1/EEI-2 was applied. We found that there is no non-linear relationship between EEI-1/EEI-2 and lung cancer in all non-smokers for either women or men (*P*_*non-linear*_ > 0.05) (Additional file 1: Table S1, Additional file 2: Figure S1).

**Table 3:** Distribution of selected environmental risk factors, the corresponding ors and 95% CIs among non-smokers

Environmental risk factors	Controls	Cases	β	aOR (95%CI)*
	(n = 767) (%)	(n = 767) (%)		
Polluting companies near home				
No	711 (92.7%)	627 (81.7%)	–	1.000
Yes	56 (7.3%)	140 (18.3%)	0.919	**2.507 (1.707, 3.681)**
Food temperature when eating (tea, soop, porridge, etc.)				
Not cold and not hot	667 (87.0%)	627 (81.7%)	–	1.000
Cold	16 (2.0%)	48 (6.3%)	1.411	**4.102 (2.035, 8.270)**
Hot	84 (11.0%)	92 (12.0%)	0.086	1.090 (0.744, 1.595)
Intake of green vegetables				
< 1/day	36 (4.7%)	75 (9.8%)	0.498	**1.646 (1.016, 2.667)**
≥ 1/day	731 (95.3%)	692 (90.2%)	–	1.000
Intake of fruit				
< 1/day	306 (39.9%)	526 (68.6%)	0.862	**2.367 (1.834, 3.055)**
≥ 1/day	461 (60.1%)	241 (31.4%)	–	1.000
Intake of seafood				
< 5/wk	725 (94.5%)	692 (90.2%)	–	1.000
≥ 5/wk	42 (5.5%)	75 (9.8%)	0.859	**2.361 (1.452, 3.841)**
Intake of fried food				
< 3/wk	749 (97.7%)	722 (94.1%)	–	1.000
≥ 3/wk	18 (2.3%)	45 (5.9%)	0.882	**2.415 (1.203, 4.849)**
Intake of smoked food				
< 3/wk	764 (99.6%)	747 (97.4%)	–	1.000
≥ 3/wk	3 (0.4%)	20 (2.6%)	1.665	**5.286 (1.292, 21.630)**
Take vitamins regularly				
No	594 (77.4%)	681 (88.8%)	0.398	**1.489 (1.063, 2.087)**
Yes	173 (22.6%)	86 (11.2%)	–	1.000
Drinking tea (years)				
Don't drink tea	487 (63.5%)	604 (78.7%)	0.764	**2.148 (1.510, 3.055)**
< 20	128 (16.7%)	68 (8.9%)	0.128	1.137 (0.707, 1.829)
≥ 20	152 (19.8%)	95 (12.4%)	–	1.000
Lifetime exposure to family’s environmental tobacco smoke (ETS) (smoker-years)^a^				
No exposure	527 (68.7%)	387 (50.5%)	–	1.000
0–28.210	135 (17.6%)	175 (22.8%)	0.632	**1.882 (1.372, 2.582)**
≥ 28.210	105 (13.7%)	205 (26.7%)	0.912	**2.490 (1.800, 3.444)**
Lifetime exposure to workplace’s environmental tobacco smoke (ETS) (years)^b^				
No exposure	636 (82.9%)	606 (79.0%)	–	1.000
0–25	66 (8.6%)	74 (9.6%)	0.223	1.250 (0.806, 1.940)
≥ 25	65 (8.5%)	87 (11.3%)	0.624	**1.867 (1.228, 2.837)**
Exercise				
No	405 (52.8%)	510 (66.5%)	0.417	**1.517 (1.186, 1.940)**
≥ 1/wk	362 (47.2%)	257 (33.5%)	–	1.000

The bold values represent *P* values less than 0.05

*aOR* adjusted odds ratio, *CI* confidence interval

^*^Adjusted for sex, age, marriage, education, occupation

^a^The indicated subgroups were categorized by the median of smoker-years in the cases and controls who were exposed to environmental tobacco smoke in family. Smoker-years at household were the product of the number of smokers smoking inside the house and the years of exposure to such behavior

^b^The indicated subgroups were categorized by the median of years in the cases and controls who were exposed to environmental tobacco smoke in workplace

Combined effects of lung disease history, environmental exposures, and family history of lung cancer on susceptibility to lung cancer.

We then evaluated the joint effects of EEI, lung disease history, and family lung cancer history (Table 4). No matter which EEI calculation method was used, the ORs of EEI-1 are equal to EEI-2. A positive association (*P*_*trend*_ < 0.001) between EEI and non-smoker lung cancer in all three groups “without family lung cancer history and without lung disease history”, “only with lung disease history”, and “only with family lung cancer history” was observed. However, owing to the sample size, few objects had lung disease history and family lung cancer history simultaneously.

**Table 4:** ORs and 95% CIs according to combined effect of lung disease history, family lung cancer history and environmental exposure index score among non-smokers

Risk factors	Without family lung cancer history				With family lung cancer history			
	Without lung disease history		With lung disease history		Without lung disease history		With lung disease history	
	Cs/cnt	OR (95%CI)	Cs/cnt	OR (95%CI)	Cs/cnt	OR (95%CI)	Cs/cnt	OR (95%CI)
EEI-1								
Low	101/345	1.000	11/24	1.493 (0.694, 3.213)	7/9	**3.597 (1.265, 10.226)**	0/2	–
Intermediate	227/247	**2.784 (2.072, 3.741)**	21/10	**7.565 (3.367, 16.998)**	12/6	**7.975 (2.829, 22.486)**	1/0	–
High	314/113	**8.163 (5.933, 11.231)**	40/8	**17.584 (7.842, 39.427)**	33/3	**35.143 (10.413, 118.602)**	0/0	–
*p* value (test for trend)	< 0.001							
EEI-2								
Low	101/345	1.000	11/24	1.493 (0.694, 3.213)	7/9	**3.597 (1.265, 10.226)**	0/2	–
Intermediate	227/247	**2.784 (2.072, 3.741**	21/10	**7.565 (3.367, 16.998)**	12/6	**7.975 (2.829, 22.486)**	1/0	–
High	314/113	**8.163 (5.933, 11.231)**	40/8	**17.584 (7.842, 39.427)**	33/3	**35.143 (10.413, 118.602)**	0/0	–
*p* value (test for trend)	< 0.001							

The bold values represent *P* values less than 0.05

ORs were adjusted for sex, age, marriage, education, occupation

*Cs/cnt* Case/Control,* OR* odds ratio, *CI* confidence interval

^#^The indicated subgroups were categorized by the tertiles of environmental exposure index (EEI) in the cases and controls

Therefore, we further analyzed the joint effects of EEI, lung disease history, and family cancer history (Table 5). We found a combined effect between lung disease history, family lung cancer history, and EEI. We did not observe a positive association between those individuals at low levels of environmental exposure who have lung disease history and family cancer history. The strongest positive gradient of associations with EEI were found among individuals with lung disease history and without family cancer history. The OR for non-smokers who had high levels of EEI and who had lung disease history was 22.306 (95%CI: 8.335–59.700), which was higher than observed for family cancer history.

**Table 5:** ORs and 95% CIs according to combined effect of lung disease history, family cancer history and environmental exposure index score among non-smokers

Risk factors^#^	Without family cancer history				With family cancer history			
	Without lung disease history		With lung disease history		Without lung disease history		With lung disease history	
	Cs/cnt	OR (95%CI)	Cs/cnt	OR (95%CI)	Cs/cnt	OR (95%CI)	Cs/cnt	OR (95%CI)
EEI-1								
Low	89/278	1.000	7/19	1.178 (0.469, 2.959)	19/76	0.960 (0.543, 1.699)	4/7	1.788 (0.497, 6.434)
Intermediate	198/209	**2.715 (1.977, 3.729)**	17/7	**7.960 (3.111, 20.367)**	41/44	**2.792 (1.684, 4.628)**	5/3	**5.916 (1.336, 26.199)**
High	253/87	**7.881 (5.547, 11.198)**	34/5	**22.306 (8.335, 59.700)**	94/29	**9.797 (5.990, 16.023)**	6/3	**6.729 (1.577, 28.718)**
*p* value (test for trend)	< 0.001							
EEI-2								
Low	89/278	1.000	7/19	1.178 (0.469, 2.959)	19/76	0.960 (0.543, 1.699)	4/7	1.788 (0.497, 6.434)
Intermediate	198/209	**2.715 (1.977, 3.729)**	17/7	**7.960 (3.111, 20.367)**	41/44	**2.792 (1.684, 4.628)**	5/3	**5.916 (1.336, 26.199)**
High	253/87	**7.881 (5.547, 11.198)**	34/5	**22.306 (8.335, 59.700)**	94/29	**9.797 (5.990, 16.023)**	6/3	**6.729 (1.577, 28.718)**
*p* value (test for trend)	< 0.001							

The bold values represent *P* values less than 0.05

ORs were adjusted for sex, age, marriage, education, occupation

*Cs/cnt* Case/Control*OR* odds ratio, *CI* confidence interval

^#^The indicated subgroups were categorized by the tertiles of environmental exposure index (EEI) in the cases and controls

We detected a statistically significant additive interaction (RPRI: 7.343, 95%CI: 0.508–14.178; AP: 0.603, 95%CI: 0.368–0.839; S: 2.920, 95%CI: 1.496–5.697) between lung disease history and the EEI, which indicated that the risk of having lung cancer was about seven-fold and the relative risk of having lung cancer was about three-fold greater than if there were no interaction. Approximately 60.3% of all non-smoker lung cancer cases were attributed to the interaction between the history of lung disease and environmental exposures. There may be an additive interaction between family lung cancer history and the EEI, considering that RPRI contained 0, but AP didn’t contain 0 and S didn’t contain 1. However, no significant multiplicative interaction was found (Table 6).

**Table 6:** Additive interaction of lung disease history, family lung cancer history and environmental exposure index score among non-smokers

Index	Lung disease history and family lung cancer history^b^	Lung disease history and EEI^a,c^	Family lung cancer history and EEI^a,d^
RERI	− 3.814 (− 8.150, 0.521)	7.343 (0.508, 14.178)^*^	11.510 (− 2.227, 25.248)
AP	− 3.138 (− 15.308, 9.033)	0.603 (0.368, 0.839)^*^	0.637 (0.327, 0.947)^*^
S	0.054 (0.000, 687, 248.783)	2.920 (1.496, 5.697)^*^	3.070 (1.206, 7.818)^*^

^*^Statistical significance

^a^EEI was dichotomous variable in this analysis, was classified as low and intermediate-high

^b^Adjusted for sex, age, marriage, education, occupation and EEI

^c^Adjusted for sex, age, marriage, education, occupation and family lung cancer history

^d^Adjusted for sex, age, marriage, education, occupation and lung disease history

## Discussion

In this case–control study on lung cancer among non-smokers, we observed the association between family history of lung cancer and susceptibility to lung cancer, and lung disease history (especially asthma) and susceptibility to lung cancer. We used two methods to develop an environmental exposure index according to 12 significant environmental factors of lung cancer. We then established restricted cubic spline model of EEI and lung cancer in non-smokers, which prompted us to conclude that there is no non-linear relationship between the two. The results from combined effects of lung disease history, family lung cancer history, and EEI score among non-smokers showed that EEI was associated with an increased risk of lung cancer and the positive association was apparently stronger in those with lung disease history or family lung cancer history. Furthermore, we found a statistically significant addictive interaction between EEI and lung disease history, as well as a possibly addictive interaction between EEI and family lung cancer history on lung cancer.

Recent studies have concluded that previous lung diseases may be linked to lung carcinogenesis in many different populations. Our research has reached a similar conclusion. Most previous studies focused on all lung cancer subjects but did not divide smokers and non-smokers. Smoking is considered a major risk factor for lung cancer and many other lung diseases. The aim of our study was restricted to non-smoker subjects to eliminate interference of lung disease as adjoint relation, which could provide a better way to identify their exact causal association with lung cancer. We also noted that previous asthma significantly increased the risk of lung cancer susceptibility in our research. A meta-analysis study [23] showed asthma was associated with the increased risk of lung cancer (OR = 1.44; 95% CI 1.31–1.59; *P* < 0.00001; *I*^2^ = 83%), but it was not associated with lung adenocarcinoma risk. Asthma is a disease of chronic airway inflammation involving multiple cells and cellular components [24]. Inflammatory mediators secreted by inflammatory cells can activate relevant protein kinase signaling pathways, regulate cell proliferation or apoptosis, induce tissue damage and repair, induce continuous cell renewal, and increase genetic mutations, thereby predisposing individuals to lung cancer. Probably, owing to the small number of subjects, we did not observe a connection between other lung disease history and lung cancer.

In the evaluation of genetic susceptibility, family history has been used as a surrogate for genetic risk [25]. Most studies have showed an increase of overall lung cancer risk for individuals with family history of lung cancer [12, 26, 27], which was inconsistent with our results. Risk assessment according to type of cancer (lung cancer and other cancer) and relatives (first-degree relatives and others) has rarely been performed in examination of non-smokers. Regarding lung cancer risk in relation to family history of cancer, the present study showed that the positive association tended to be evident for family history of lung cancer, family history of other cancer (except lung cancer), and family history of total cancer in first-degree relatives yet was not associated with the risk of lung cancer in non-smokers. Our conclusions regarding lung cancer susceptibility in relation to family history of other cancers were inconsistent with some previous studies. A study [26] from Japan indicated history of breast cancer in siblings was positively associated with the risk of overall lung cancer, small cell carcinoma, and adenocarcinoma among men. However, another Japanese study [28] reported that women with a family history of breast cancer in first-degree relatives are at higher risk of adenocarcinoma than men. Our study did not collect specific classification of other cancers in non-smokers, so we did not cover this issue in a more in depth. Further studies of the type of family cancer history and lung cancer are required.

Our results also implied the importance of shared exposure to environmental and residential factors in the development of lung cancer. Among the significant environmental risk factors in present study, intake of smoked food ≥ 3/wk showed the strongest effects on lung cancer, which was similar to our previous research result [29]. Food would likely be contaminated to a certain extent when it was smoked at high temperatures and the content of carcinogen (i.e., Benzo-a-pyrene [B(a)P]) in food would increase greatly. Extensive in vitro and in vivo investigations have demonstrated the capability of B(a)P to impose a variety of lethal factors capable of instigating and/or promoting the multi-step lung carcinogenesis process [30]. B(a)P can cause mutations in crucial genes by binding to DNA, which can contribute to the conversion of normal cells into tumor cells. B(a)P is also capable of promoting the outgrowth of transformed cells and contributing to the generation of visible tumor cell masses. A study [31] of barbecued meat, which also can accumulate B(a)P, demonstrated a positive association with risk of lung cancer. This emphasizes the importance of the cooking method of food in lung cancer.

To improve statistical efficiency, we integrated multiple environmental factors into a unitary environment exposure index to evaluate the overall influence of environmental exposure. These two methods were based on β and OR, respectively, and were used to calculate EEI score. Additionally, we applied restricted cubic spline that provided a useful and flexible basis for modeling relationships among continuous predictors to analyze the non-linear relationship between EEI and lung cancer in non-smokers. Since the calculation formula of OR is related to β, the present result revealed EEI was not non-linear-related with lung cancer in women and men, and the increase of environmental exposure factors appears to be associated with an increased risk of lung cancer using two different EEI scores. Therefore, an individual who was exposed to more environmental factors of high-risk rating would have a higher risk of lung cancer.

Although some previous studies have explored the influence of environmental factors in lung cancer, few illuminated the combined effects of lung disease history, environmental exposures, and family history of lung cancer among non-smokers. After adjusting general demographic factors, our environmental exposure index showed a positive gradient association with lung cancer whether for those “without family lung cancer history and lung disease history”, “only with lung disease history”, or “only with family lung cancer history”. Moreover, no matter what level of EEI was used, family history of lung cancer was a higher risk of developing lung cancer than lung disease history for non-smokers. However, non-smokers who had lung disease history were at higher risks of lung cancer than non-smokers who had family cancer history, especially for those who had intermediate-high levels of EEI. This is consistent with the above conclusion that family history of other cancers is not associated with the risk of lung cancer in non-smokers. Furthermore, there was an addictive interaction between EEI and lung disease history/family lung cancer history on lung cancer. Therefore, the present study indicated that environmental exposure had a combined effect with lung disease history or family history of lung cancer, which suggested that environmental factors and lung disease history or environmental factors and family history of lung cancer might interact with each other and synergistically increase the risk of developing lung cancer.

We do acknowledge that our study has several limitations. The first limitation was that the sample size may not have been sufficient, which resulted in few objects classified with family history of lung cancer and lung disease history. However, we are continuing to collect cases and controls to expand the sample size. Second, the present research was a hospital-based case–control study and the potential selection bias and recall bias could not be completely avoided. Third, we mainly focused on environment-disease history-family history and did not explore genetic susceptibility in this study. Family history of lung cancer is a characteristic of inherited susceptibility in lung cancer and cannot be completely used as a surrogate for genetic risk. Fourth, occupational exposures were an important cause of lung cancer as previously reported [32], but our research was inadequate in this respect since having the difficulty of using job-exposure matrix (JEM) to estimate the individual exposures in our country. We had difficulty to assigning individual exposure to a specific carcinogen based on job title only due to irregular production processes and operations for many small factories and workers’ work types which were not fixed. Although we have used using occupation as an adjustment factor to control the confounding effects of occupational exposure to a certain extent in our study, further studies will be needed to explore the mechanism of occupational carcinogens on lung cancer.

## Conclusions

It is important to recognize the etiology and potential mechanism of non-smoker lung cancer. In this case–control study on lung cancer among non-smokers, we observed the association between family history of lung cancer and susceptibility to lung cancer, and lung disease history (especially asthma) and susceptibility to lung cancer. There were combined effects among lung disease history, environmental exposures, and family history of lung cancer toward susceptibility to lung cancer in Chinese non-smokers. Non-smokers who had a family history of lung cancer were at higher risk of lung cancer than non-smokers who had lung disease history. Furthermore, there was a statistically significant addictive interaction between EEI and lung disease history, and a possibly addictive interaction between EEI and family lung cancer history on development of lung cancer. Non-smokers with family cancer history may obtain benefits from removal of environmental exposures and active treatment of lung disease.

## Supplementary Information

**Additional file 1: Table S1.** Restricted Cubic Spline Model of EEI and Lung Cancer, the Corresponding *β*, *AIC* and *R*^2^ Among Non-Smokers

**Additional file 2: Figure S1.** Restricted cubic spline model of EEI and lung cancer in non-smokers.

**Additional file 3. **Briefly describe the questionnaire.

## Data Availability

The datasets used and analysed during the current study are available from the corresponding author on reasonable request.

## Abbreviations

AP: The attributable proportion due to interaction
B(a)P: Benzo-a-pyrene
CI: Confidence interval
COF: Cooking oil fumes
COPD: Chronic obstructive pulmonary disease
EEI: Environmental exposure index
ETS: Environmental tobacco smoke
OR: Odds ratio
PM: Particulate matter
RCS: Restricted cubic spline
RERI: The relative excess risk due to interaction
S: The synergy index
TB: Tuberculosis
