# Supplementary material for: Combined effects of lung disease history, environmental exposures, and family history of lung cancer to susceptibility of lung cancer in Chinese non-smokers
Source: Respir Res. 2021 Jul 23;22:210. doi: 10.1186/s12931-021-01802-z (PMC8306005; doi:10.1186/s12931-021-01802-z)
Supplement: Supplementary file 1 — Additional file 1: Table S1. Restricted Cubic Spline Model of EEI and Lung Cancer, the Corresponding β, AIC and R2 Among Non-Smokers [file 12931_2021_1802_MOESM1_ESM.docx]

Additional file 1: Table S1 Restricted Cubic Spline Model of EEI and Lung Cancer, the Corresponding *β*, *AIC* and *R*^2^ Among Non-Smokers

|  | All^a^ | Female^b^ | Male^b^ |
| --- | --- | --- | --- |
| EEI-1 |  |  |  |
| *β*_1_ (95%*CI*) | 0.742(0.366,1.117) | 0.833(0.448,1.219) | 0.644(-0.205,1.492) |
| *p* | <0.001 | <0.001 | 0.137 |
| *β*_2_ (95%*CI*) | 0.207(-1.135,1.549) | 0.032(-1.451,1.516) | -0.157(-3.126,2.811) |
| *p* | 0.763 | 0.966 | 0.917 |
| *β*_3_ (95%*CI*) | -1.067(-4.170,2.036) | -0.801(-4.137,2.534) | 0.227(-6.729,7.183) |
| *p* | 0.500 | 0.638 | 0.949 |
| Akaike crit (*AIC*) | 1731.718 | 1360.295 | 384.187 |
| Pseudo *R*^2^ | 0.196 | 0.213 | 0.141 |
| EEI-2 |  |  |  |
| *β*_1_ (95%*CI*) | 0.120(0.063,0.176) | 0.131(0.070,0.192) | 0.111(-0.009,0.231) |
| *p* | <0.001 | <0.001 | 0.071 |
| *β*_2_ (95%*CI*) | -0.009(-0.194,0.176) | -0.019(-0.250,0.212) | -0.074(-0.469,0.321) |
| *p* | 0.923 | 0.870 | 0.713 |
| *β*_3_ (95%*CI*) | -0.043(-0.511,0.424) | -0.034(-0.549,0.482) | 0.175(-0.828,1.178) |
| *p* | 0.855 | 0.898 | 0.732 |
| Akaike crit (*AIC*) | 1729.527 | 1357.957 | 384.775 |
| Pseudo *R*^2^ | 0.197 | 0.214 | 0.140 |

^a^ were adjusted for gender, age, marriage, education, occupation, lung disease history and family lung cancer history.

^b^ were adjusted for age, marriage, education, occupation, lung disease history and family lung cancer history.
