# Supplementary material for: Combined effects of lung disease history, environmental exposures, and family history of lung cancer to susceptibility of lung cancer in Chinese non-smokers
Source: Respir Res. 2021 Jul 23;22:210. doi: 10.1186/s12931-021-01802-z (PMC8306005; doi:10.1186/s12931-021-01802-z)
Supplement: Supplementary file 2 — Additional file 2: Figure S1. Restricted cubic spline model of EEI and lung cancer in non-smokers. [file 12931_2021_1802_MOESM2_ESM.docx]

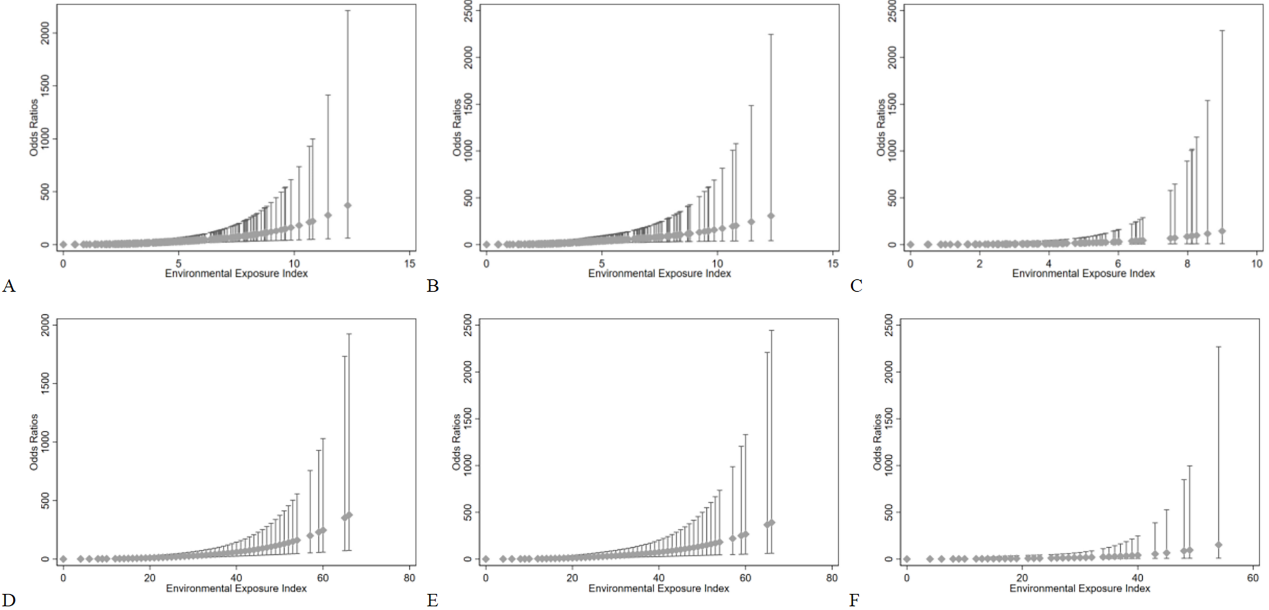


Additional file 2: Figure S1. Restricted cubic spline model of EEI and lung cancer in non-smokers. (A) Using EEI-1 in all non-smokers. (B) Using EEI-1 in female. (C) Using EEI-1 in male. (D) Using EEI-2 in all non-smokers. (E) Using EEI-2 in female. (F) Using EEI-2 in male.
