# Supplementary material for: Combined effects of lung disease history, environmental exposures, and family history of lung cancer to susceptibility of lung cancer in Chinese non-smokers
Source: Respir Res. 2021 Jul 23;22:210. doi: 10.1186/s12931-021-01802-z (PMC8306005; doi:10.1186/s12931-021-01802-z)
Supplement: Supplementary file 3 — Additional file 3. Briefly describe the questionnaire. [file 12931_2021_1802_MOESM3_ESM.docx]

**Briefly describe the questionnaire**

The questionnaire used in this study is divided into 7 parts: informed consent, sociodemographic information, living environment, diet, environmental tobacco smoke, physical activity, disease history and family history of tumors.

1)Informed consent: Inform objects of the purpose, significance, and content of the survey. Hope to get the support and cooperation of participants. We will keep strictly confidential for the results of the investigation. Please sign the informed consent form after knowing.

2)Sociodemographic information: included name, gender, age, visited hospital, family address, date of birth, ethnicity, education level, marital status, and family members.

3)Living environment: Is the current residence in rural or urban area? Are there polluting companies or factories near your long-term residence? Is there a irritant smell in the house after renovations, and how long does it last? Is an exhaust fan or range hood installed in the kitchen? Do you like to burn the oil very hot when you cooking by yourself? How often do you fry food by yourself?

4)Diet: included frequency of food consumption and eating habits. The food intake surveyed are green vegetable, fruit, meat, fish, seafood, dairy products, bean products, eggs, pickled food, fried food, smoked food, and vitamin. The eating habits included food temperature when eating (tea, soop and porridge, etc), habit of eating garlic raw, drinking history and tea drinking history. Drinking alcohol is defined as at least 1 time/week and for more than half a year. Drinking tea is defined as at least 1 cup/week and for more than half a year.

5)Environmental tobacco smoke (ETS): ETS is defined as non-smokers smoke at least one day (more than 15 minutes per day) of inhaled cigarettes or smoke exhaled by smokers. Investigated the number of smokers in the household/workplace and the time spent with them.

6)Physical activity: Investigated whether objects exercise and frequency of exercise.

7)Disease history and family history of tumors: Disease history mainly investigated the history of lung disease, including chronic bronchitis, tuberculosis, pneumonia, chronic obstructive pulmonary disease, asthma, pneumoconiosis, silicosis and others. Family history of tumors: Who suffers from cancer? What kind of relationship? What type of cancer?
